# Positive association between physical outcomes and patient-reported outcomes in late-onset Pompe disease: a cross sectional study

**DOI:** 10.1186/s13023-020-01469-7

**Published:** 2020-09-03

**Authors:** Meng Yuan, Eleni-Rosalina Andrinopoulou, Michelle E. Kruijshaar, Aglina Lika, Laurike Harlaar, Ans T. van der Ploeg, Dimitris Rizopoulos, Nadine A. M. E. van der Beek

**Affiliations:** 1 Department of Biostatistics, Erasmus MC University Medical Center, Rotterdam, The Netherlands; 2 Department of Pediatrics, Center for Lysosomal and Metabolic Diseases, Erasmus MC University Medical Center, Rotterdam, The Netherlands; 3 Department of Neurology, Center for Lysosomal and Metabolic Diseases, Erasmus MC University Medical Center, Rotterdam, The Netherlands

**Keywords:** Late-onset Pompe disease, Patient-reported outcome measures, Muscle strength, 6-min walk test, Forced vital capacity

## Abstract

**Background:**

Pompe disease is a rare, progressive metabolic myopathy. The aim of this study is to investigate the associations of physical outcomes with patient-reported outcome measures (PROMs) in late-onset Pompe disease.

**Methods:**

We included 121 Dutch adult patients with Pompe disease.

Physical outcomes comprised muscle strength (manual muscle testing using Medical Research Council [MRC] grading, hand-held dynamometry [HHD]), walking ability (6-min walk test [6MWT]), and pulmonary function (forced vital capacity [FVC] in upright and supine positions).

PROMs comprised quality of life (Short Form 36 health survey [SF-36]), participation (Rotterdam Handicap Scale [RHS]) and daily-life activities (Rasch-Built Pompe-Specific Activity [R-PAct] Scale).

Analyses were cross-sectional: the time-point before, and closest to, start of Enzyme Replacement Therapy was chosen. Associations between PROMs and physical outcomes were investigated using linear regression models.

**Results:**

RHS and R-PAct scores were better in patients with higher FVC supine and upright, HHD, MRC and 6MWT scores, accounting for the effect of sex, disease duration, use of wheelchair and ventilator support. While the SF-36 Physical Component Summary (PCS) was correlated positively with FVC upright, HHD, MRC and 6MWT scores, there was no significant relationship between the SF-36 Mental Component Summary (MCS) and any of the physical outcomes.

**Conclusions:**

Participation, daily-life activities, and the physical component of quality of life of adult Pompe patients are positively correlated to physical outcomes. This work serves as a first step towards assessing how changes over time in physical outcomes are related to changes in PROMs, and to define the minimal change in physical outcomes required to make an important difference for the patient.

## Background

Pompe disease is a rare, progressive metabolic myopathy. Partial or total deficiency of the enzyme acid *α*-glucosidase leads to a build up of lysosomal glycogen, causing cellular damage in virtually all body tissues, and in particular muscle [1]. Patients with late-onset Pompe disease present with a progressive muscle weakness, which leads to limitations in motor function and respiratory difficulties. Since 2006, Enzyme Replacement Therapy (ERT) with recombinant human alpha-glucosidase has been available for Pompe disease.

Many studies have investigated the effects of ERT in adult patients with Pompe disease. After the initial placebo-controlled trial [2], studies were observational and mostly without a control group comparing to the baseline situation before treatment started [3–8]. A few studies also compared to patients’ expected disease course had they remained untreated [9, 10]. Together these studies have provided evidence of a beneficial effect of ERT at group level on physical outcomes (motor performance, muscle strength, pulmonary function), and survival [11, 12]. At individual patient level, the response to treatment varied. The improvements in physical outcomes were found to be greatest in the first 2 to 3 years after ERT, but also after 5 years of treatment these outcomes were better at group level than expected without treatment [10]. Follow-up of 30 patients from the initial trial showed large variation in the response after 10 years, with 52% having a similar or better distance walked and/or pulmonary function compared to when they started treatment [13].

An important question is how patients experience their disease and the effects of ERT. A few studies have assessed patient reported outcome measures (PROMs) in Pompe disease. These have shown that the disease has a large impact on the participation of patients in daily life, and that the physical domains of their quality of life are affected but the mental domains less so [2, 14, 15]. With ERT, participation stabilised at group level, the physical domains of quality of life improved initially and then remained stable, while the mental domains of quality of life did not change before and during ERT [16].

PROMs and physical outcomes thus both sketch a picture of a progressive disease and a positive effect of ERT at group level. However, it is unknown how well the physical outcome measures reflect what an individual patient experiences, and whether changes over time in physical outcomes capture changes in PROMs for an individual patient. Given that physical outcomes are more often assessed than PROMs, it would be useful to know how much benefit a patient experiences (in terms of their PROMs) from an improvement in a physical outcome, and how large a change in physical outcomes needs to be to result in a meaningful improvement in the experience of a patient. To assess how well these measures coincide for an individual patient, PROMs and physical outcomes need to be collected in the same patients at similar time-points.

Here we report on the first comprehensive study to investigate the associations of physical outcomes with PROMs cross-sectionally. Data from the International Pompe Association (IPA)/Erasmus MC survey [17], which collects several PROMs in an international group of patients, was used. For Dutch patients this was linked to the most commonly reported physical outcomes. We modelled the relationship between physical outcomes and PROMs at a specific point in time when the patient was not yet treated.

## Methods

### Data collection

This study was performed as part of two prospective observational cohort studies in Dutch patients with a confirmed diagnosis of Pompe disease. The studies are conducted at the Center for Lysosomal and Metabolic Diseases, Erasmus MC University Medical Center, Rotterdam, the national referral center for Pompe disease in the Netherlands. Physical outcomes were assessed every 3 to 6 months before and after the start of ERT since January 2005 [9]. PROMs were collected through questionnaires, which were mostly sent out through the IPA/Erasmus MC Pompe survey. This annual survey was sent out by mail from May 2002 onwards, and since May 2009 also collected through a secure web survey system [17]. The database for the current study was locked in December 2018. It included 121 Dutch adult patients. Both studies were approved by the ethics committee of the Erasmus MC University Medical Center and written informed consent was obtained from all participants.

### Outcome measures

#### Clinical outcome measures

Skeletal muscle strength was measured using the Medical Research Council (MRC) grading scale and by hand-held dynamometry (HHD; Cytec dynamometer, Groningen, The Netherlands) [18, 19]. The following muscle groups were tested for both methods: neck extensors, neck flexors, shoulder abductors, elbow flexors, elbow extensors, hip flexors, hip abductors, knee flexors, and knee extensors. In addition, the MRC grade was determined for the shoulder adductors, shoulder exorotators and endorotators, hip extensors, and hip adductors. This was expressed as the percentage of the maximum possible score for MRC sum scores, and as the percentage of the median strength of healthy males and females for HHD sum scores. In the present analysis, no score was calculated if three or more items were missing. Walking ability was assessed using the six-minute walk test (6MWT) in which the distance walked in 6 min was recorded [20]. The results were presented as a percentage of the predicted normal values [21]. Forced vital capacity (FVC) was measured in upright seated and supine positions. Results were expressed as the percentage of the predicted FVC [22, 23]. The use of wheelchair and ventilator support was registered at each visit.

#### Patient-reported outcome measures (PROMs)

Quality of life was assessed using the Medical Outcome Study 36-item Short Form Health Survey (SF-36) [24]. Summary scores in two domains were derived: the physical component summary measure (PCS) and the mental component summary measure (MCS). Norm-based scores were calculated using the Dutch 1998 norm-based scoring, ensuring the comparability of the results for both versions of the SF-36 [25]. (SF-36 version 1.0 for 2002 to 2009, and afterwards version 2.0).

Participation in daily life activities was assessed using the Rotterdam Handicap Scale (RHS) [15, 26]. Ability to carry out daily life activities was assessed by using the Rasch-Built Pompe-Specific Activity (R-PAct) scale [27]. The RHS is a general measurement scale, which comprises 9 items. The scores per item range from 1 (‘unable to fulfil the task or activity’) to 4 (‘complete fulfilment of the task or activity’). A score of 0 indicated that the task or activity could not be applied. The total score is calculated as the sum of the item scores divided by the number of applicable items and multiplied by 9. Sum scores were calculated if at most three items were non-applicable or missing. The R-PAct scale is designed specifically for Pompe disease, which consists of 18 items addressing daily life activities with three response options (0 = unable to perform; 1 = able to perform, but with difficulty; 2 = able to perform without difficulty). Only when all items had been answered, a centile metric score (0–100) was calculated as described previously [27].

### Statistical analysis

Continuous variables are presented as median and range while categorical variables are presented as frequency and percentage.

The physical measurements and the PROMs were collected repetitively over time in both cohort studies. Also before ERT there were usually multiple measurements available. We selected only those pairs of physical outcome and PROM that were less than 3 months apart (and before start of ERT). When multiple pairs were available for a patient, we chose the pair that was closest to the start of ERT. The number of patients for each pair of outcomes are presented in the Supplement, Table 1.

The relationship between each physical outcome and each patient-reported outcome was investigated using a linear regression model, which included sex, disease duration and use of wheelchair and ventilator as potential confounders. We calculated disease duration as the difference between the date of symptom onset and the date the physical outcome was assessed.

The first step to build the model was to investigate whether the association between physical outcomes and PROMs is linear or non-linear. We assumed a flexible non-linear relationship using smoothing spline functions for the physical measurement variables, natural cubic splines with up to 2 degrees of freedom. The F-test was used to investigate which of the models (linear versus non-linear) was more appropriate.

A problem that arises when comparing the results between models with different outcomes is that the outcomes are not measured on the same scale. To overcome this, we present the standardized regression coefficients in addition to the original coefficients. Each variable can be standardized by subtracting its mean from each of its values and then dividing by the standard deviation of the variable prior to the regression analysis. The standardized coefficients can then be interpreted as the number of standard deviations the dependent outcome will change if the independent outcome changes one standard deviation.

The multiple testing problem was addressed by the Holm method and a significance level of 0.05 was assumed. Models were fitted for each pair of outcome measures using the statistical program R (version 3.5.1) (ref: R Core Team (2019). R: A language and environment for statistical computing. R Foundation for Statistical Computing, Vienna, Austria. URL http://www.R-project.org/).

## Results

### Participants

**Fig. 1:**
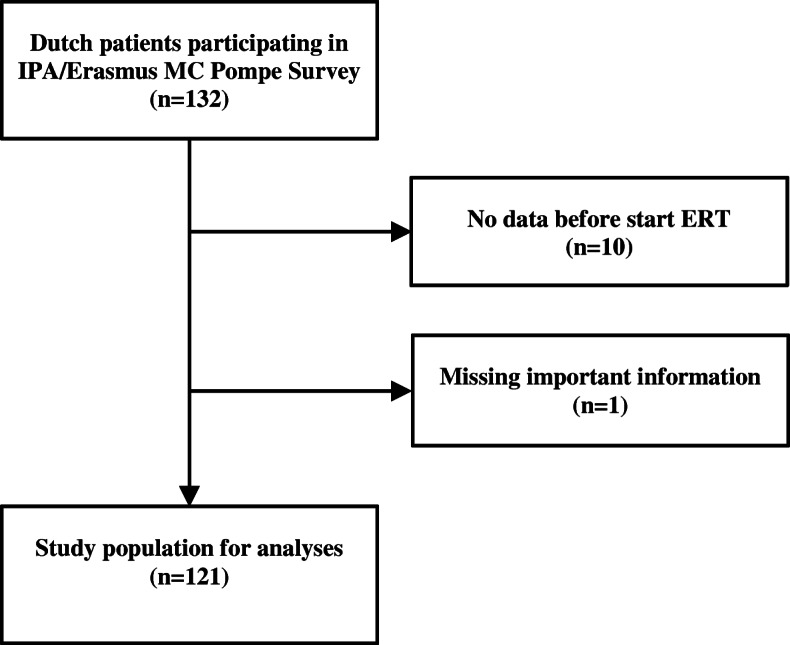
Flowchart of the study population for analyses

One hundred and twenty-one adult Pompe patients were included for this study. The inclusion process was as shown in Fig. 1. There were two reasons for excluding patients: no PROMs collected before the start of ERT (*n* = 10) and no record of date of symptom onset available (*n* = 1). For each pair of physical outcome and PROM that we analyzed, the number of patients available for analysis was determined by whether these outcomes were assessed before ERT and whether the two measurements were within 3 months of each other. The exact number of patients per analysis can be found in the Supplement Table 1.

Table 1 shows the characteristics of the study population at the time of examination. Participants had a median age of 53 years (range 23–79) and a median disease duration of 16 years (range 0.2–50), 53% were women, 45% were partially or permanently wheelchair dependent, and 26% used mechanical ventilation.

**Table 1:** Characteristics of the study population

**Demographic and clinical characteristics**	**Patients (*n*** **= 121)**
Women: number (%)	64 (53)
Age at examination in years: median (range)	53 (23–79)
Disease duration^a^ at examination in years: median (range)	16 (0.2–50)
Wheelchair^b^ use at examination: number (%)	54 (45)
Respiratory support at examination: number (%)	32 (26)
**Physical outcomes**	**Median (range)**
FVC upright (% pred)	77 (10–117)
FVC supine (% pred)	63 (13–107)
HHD sum score (% max)	81 (21–100)
MRC sum score (% max)	85 (39–100)
6MWT (% pred)	67 (6–120)
**PROMs**	**Median (range)**
SF-36 PCS score (norm-based)	33 (17–63)
SF-36 MCS score (norm-based)	48 (19–72)
RHS score	29 (11–36)
R-PAct score	60 (7–100)

*FVC* Forced Vital Capacity, *HHD* Hand-Held Dynamometry, *MRC* Medical Research Council, *6MWT* Six-Minute Walk Test, *SF-36* Short-Form 36 Health Survey, *RHS* Rotterdam Handicap Scale, *R-Pact* Rash-built Pompe-Specific Activity scale

^a^duration from symptom onset

^b^partial and permanent wheelchair use

### Associations between physical outcomes and PROMs

First, we assessed whether linear or non-linear models were needed to describe relationships between the physical outcomes and PROMs. In most cases, non-linear models did not provide a better fit of the data, and linear models were selected. For the relationship between the MRC and PCS, and the HHD and R-PAct non-linear models were statistically better (*p*-values are presented in the Supplement Table 2). However, on visual inspection of these two distributions, there is no strong evidence supporting the non-linear models. The Supplement Fig. 1 displays the fitted results of linear and non-linear models for both distributions. For simplicity, the linear models were therefore used.

In the two following sections, we will use two pairs of outcomes as examples to describe the interpretation of the results. In the clinical trial on ERT in Pompe disease, the two key physical outcomes were FVC upright and 6MWT scores. We will describe the positive relationships of FVC upright with RHS, and of 6MWT with R-Pact below. The results for all models can be found in the Supplement material Table 3.1–3.20.

### FVC upright with RHS

On average across patients, scoring 1 %-point higher on the FVC upright (presented as percentage of predicted) corresponded to a 0.147 points higher RHS score (Estimate: 0.147; 95%CI [0.107, 0.186]; *p* < 0.001), accounting for sex, disease duration and the use of wheelchair and ventilator (Supplement Table 3.3).

Also wheelchair dependence (Estimate: -4.069; 95%CI [− 5.599, − 2.539]) was associated with a worse RHS score: a wheelchair dependent person having a 4.069 point lower RHS score on average. No significant difference in RHS scores was found among patients of different sex, disease duration and the use of ventilation (Supplement Table 3.3).

**Fig. 2:**
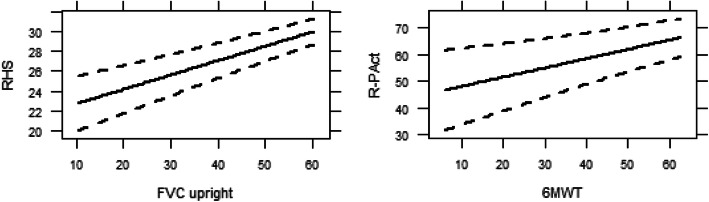
Modelled results for the relationship between FVC upright and RHS (left), and 6MWT and R-PAct (right) for female patients with median disease duration (16 years) and without wheelchair and respiratory support

Figure 2 (left-hand side) shows the model results for the relationships between FVC upright and RHS in the situation where the patient is female, does not use a wheelchair or respiratory support and has the population’s median disease duration of 16 years.

### 6MWT with R-PAct

A 1 %-point higher 6MWT (also presented as percentage of predicted) corresponded, on average, to a 0.348 unit higher R-PAct score (Estimate: 0.348; 95%CI [0.147, 0.549]; *p* = 0.01), accounting for sex, disease duration and the use of wheelchair and ventilator (Supplement Table 3.20).

R-PAct scores were not found to be related to sex, disease duration and the use of wheelchair and ventilation (Supplement Table 3.20). Figure 2 (right-hand side) shows the model results for the relationships between 6MWT and R-PAct for a female patients with a median disease duration and no wheelchair or respiratory support.

### Strength of associations across models

**Fig. 3:**
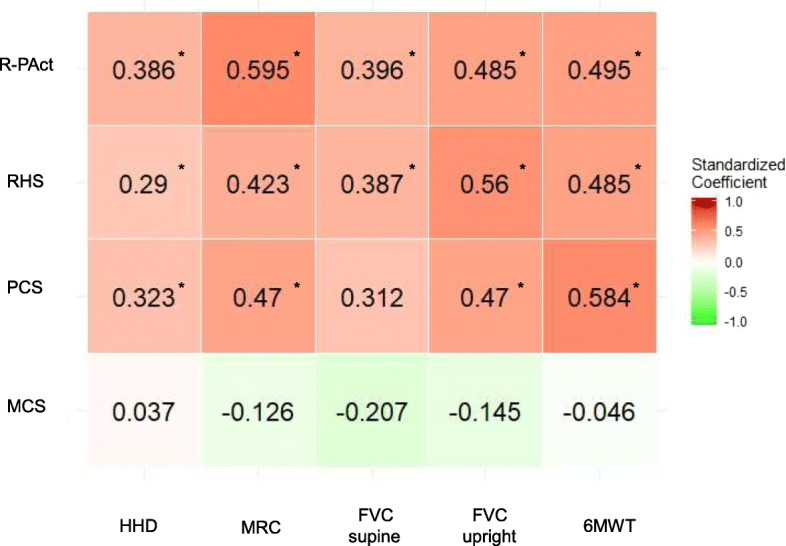
Associations between physical outcomes and PROMs. Heatmap visualizing the strength of the associations between physical outcomes and PROMs. Each cell represents one linear regression model. The standardized regression coefficient are displayed to ensure the comparability of different models. Green indicates a negative association, red a positive one. The stronger the association, the more intense the color. Statistically significant coefficients are indicated by *. Detailed results including estimates for sex, disease duration and the use of wheelchair and ventilator can be found in the supplemental material

The aforementioned results provide the interpretation for the specific associations between one pair of physical outcome and PROM, using the original regression coefficients. However, to compare the strength of the relationships across models, we applied standardized regression coefficients. Please note that these standardized coefficients do not have the same interpretation as the original coefficients. They do not correspond to the unit of measurement of the specific outcomes, and can therefore be compared in size between the different pairs of outcomes. Figure 3 provides the visualization of the strength of the associations as a heatmap. Green indicates a negative association, red positive. The stronger the association, the more intense the color.

A better FVC upright was associated with better PCS scores (Standardized Estimate: 0.47; CI [0.244, 0.696]), RHS scores (Standardized Estimate: 0.56; 95%CI [0.41, 0.71]) and R-PAct scores (Standardized Estimate: 0.485; 95%CI [0.304, 0.666]), accounting for the effect of sex, disease duration and the use of wheelchair and ventilator. Higher FVC supine score was associated with better RHS (Standardized Estimate: 0.387; 95%CI [0.207, 0.567]) and R-PAct (Standardized Estimate: 0.396; CI [0.203, 0.588]), accounting for the effect of sex, disease duration and the use of wheelchair and ventilator. A borderline significant relationship was found between the PCS score and FVC supine (*p* = 0.061, Table 4 in the supplementary material), and, in general, the standardized coefficients for FVC upright were larger than for FVC supine.

The MRC score was positively correlated with the PCS (Standardized Estimate: 0.47; 95%CI [0.254, 0.686]), RHS (Standardized Estimate: 0.423; 95%CI [0.262, 0.583]) and R-PAct (Standardized Estimate: 0.595; 95%CI [0.426, 0.764]), accounting for the effect of sex, disease duration and the use of wheelchair and ventilator. The HHD score was positively correlated with the PCS (Standardized Estimate: 0.323; 95%CI [0.096, 0.55]), RHS (Standardized Estimate: 0.29; 95%CI [0.105, 0.474]) and R-PAct (Standardized Estimate: 0.386; 95%CI [0.196, 0.577]), accounting for the effect of sex, disease duration and the use of wheelchair and ventilator. In comparison, HHD standardized coefficients were smaller than for MRC.

The 6MWT score was positively associated with the PCS (Standardized Estimate: 0.584; 95%CI [0.289, 0.878]), RHS (Standardized Estimate: 0.485; 95%CI [0.232, 0.739]) and R-PAct (Standardized Estimate: 0.495; 95%CI [0.217, 0.773]), accounting for the effect of sex, disease duration and the use of wheelchair and ventilator.

No significant relationships were found between the mental component of the SF36 and any of the physical outcome measures (*p* > 0.05, Table 4 in the supplementary material).

The size of the standardized coefficients can be interpreted in terms of standard deviations. In bird-view, the R-PAct, RHS and PCS would be between 0.3 and 0.6 (rounded) standard deviations higher when a physical outcome is one standard deviation larger.

## Discussion

This is the first study to comprehensively evaluate the associations of PROMs with physical outcomes in adult patients with Pompe disease. We show that participation in, and the ability to perform, daily life activities were positively correlated with all physical outcomes. Also, the physical component of the SF-36 (PCS) was positively correlated with the physical outcome measures, except that there was only a borderline significant association with FVC supine. No significant relationships were found between the SF-36 mental scores (MCS) and any of the physical outcome.

Two earlier studies have looked at associations between one PROM and one or two physical outcome(s). The present study on the other hand gives a comprehensive overview as it includes the most commonly assessed PROMs and clinical outcomes. In an earlier study from our own group we had already reported the positive associations between participation in daily life (RHS) and the FVC and MRC [28]. Positive associations of the physical component of quality of life (PCS) with both FVC upright and the MRC were also described in an international meta-analysis [29]. This study corroborates the present results, as the individual level data used in the international study did not include the Dutch patients included in the present study.

The absence of a relationship between the physical outcomes and MCS may suggest that the differences in physical outcomes were not associated with changes in mental health. Also in previous studies of Pompe patients, mental health scores were found to be within the normal range and no improvement was seen with ERT [14, 16]. An explanation might be that late-onset Pompe disease is a chronic disease and affects patients progressively. As a result, patients may adapt to their situation over time and adjust their expectations [30], explaining the normal mental health scores. This phenomenon is called response shift, and is a recognized issue in studies of health-related quality of life amongst chronic patients, including cancer patients [31, 32].

For each pair of physical outcome and PROM, the number of patients available for analysis was different. There were slightly more pairs of PROMs with upright FVC than with supine FVC, as more severely affected patients with Pompe disease may not be able to perform lung function testing in supine position, while they can still be tested in upright position. Possibly, the slightly lower number of measurements explains the borderline *p*-value that was observed for the association between FVC supine and PCS. The fact that more severely affected patients may be excluded from supine FVC testing also means that for PROMs paired with FVC supine the range of measured outcome values (both PROM and FVC) may be narrower than for the pairing with FVC upright. Together with the somewhat lower number of measurements, this might explain why the association of PROMs with supine FVC was less strong than with upright FVC.

A similar difference was found for muscle strength, where the strength of associations with PROMs were found to be larger for MRC scores compared to HHD scores. Here, the reason could be that more muscle groups were measured using MRC grading scale. Another reason could be there are more missing values for the HHD scores (as shown in Table A1). The number of patients for whom 6MWT data were available before ERT was smaller than all other outcomes. While reasons for this include that severely affected patients may not be able to perform this test, the main reason for this was that the test was not yet a standard outcome measure in the first years of this study.

Our analyses are based on 121 adult Pompe patients, which is a very large sample size for this rare disease. However, for statistical analyses this number was still limited and meant that the number of confounders corrected for was the maximum possible. Another concern may be that the number of pairs available for each analyses may not differ at random but are driven by disease severity. This is true for the smaller number of supine FVC measurements compared to upright, and partly for the 6MWT, and the interpretation of these results should be limited to exclude the most severe patients. However, the differences between upright and supine measurements available were not large, and the fewer 6MWT assessments available was mostly random (calendar time).

### Clinical implications

The positive associations observed suggest that physical outcomes reflect at least part of what the patient experiences. The next question to ask is how much PROMs improve when a patient’s physical outcome improves. For example, if the patient can walk 10 m extra on the 6MWT, can he/she do the grocery shopping alone? Ultimately, we would like to be able to give guidance on how much a physical outcomes needs to improve to make an important difference for the patient with Pompe disease.

## Conclusions

Here we confirm that for adult Pompe patients who are not yet treated with ERT, there is a relationship between physical outcomes and PROMs at the individual patient level, except for the mental aspect of quality of life. This study serves as a first step towards disentangling the relations between physical outcomes and PROMs over time and towards providing guidance on how much of a change is meaningful for the patient. The next steps in this research will therefore be to study the associations between these sets of outcomes over time and with ERT and to define the minimal clinically important difference (MCID) under ERT for Pompe disease that can be used for clinical decision making.

## Supplementary information

**Additional file 1.**

## Data Availability

The data that support the findings of this study are not publicly available to protect patient privacy. Please contact the author (NvdB) for further information.

## References

[1] Hirschhorn R, Reuser AJ, Scriver CR, Beaudet AL, Valle D, Sly W, Childs B, Kinzler KW (2001). Glycogen storage disease type II: acid alpha-glucosidase (acid maltase) deficiency. The metabolic and molecular bases of inherited disease.

[2] van der Ploeg AT, Clemens PR, Corzo D, Escolar DM, Florence J, Groeneveld GJ (2010). A randomized study of alglucosidase alfa in late-onset Pompe’s disease. N Engl J Med.

[3] Anderson LJ, Henley W, Wyatt KM, Nikolaou V, Waldek S, Hughes DA (2014). Effectiveness of enzyme replacement therapy in adults with late-onset Pompe disease: results from the NCS-LSD cohort study. J Inherit Metab Dis.

[4] Angelini C, Semplicini C, Ravaglia S, Bembi B, Servidei S, Pegoraro E (2012). Observational clinical study in juvenile-adult glycogenosis type 2 patients undergoing enzyme replacement therapy for up to 4 years. J Neurol.

[5] Bembi B, Pisa FE, Confalonieri M, Ciana G, Fiumara A, Parini R (2010). Long-term observational, non-randomized study of enzyme replacement therapy in late-onset glycogenosis type II. J Inherit Metab Dis.

[6] Regnery C, Kornblum C, Hanisch F, Vielhaber S, Strigl-Pill N, Grunert B (2012). 36 months observational clinical study of 38 adult Pompe disease patients under alglucosidase alfa enzyme replacement therapy. J Inherit Metab Dis.

[7] Strothotte S, Strigl-Pill N, Grunert B, Kornblum C, Eger K, Wessig C (2010). Enzyme replacement therapy with alglucosidase alfa in 44 patients with late-onset glycogen storage disease type 2: 12-month results of an observational clinical trial. J Neurol.

[8] Van der Ploeg AT, Barohn R, Carlson L, Charrow J, Clemens PR, Hopkin RJ (2012). Open-label extension study following the late-onset treatment study (LOTS) of alglucosidase alfa. Mol Genet Metab.

[9] de Vries JM, van der Beek NA, Hop WC, Karstens FP, Wokke JH, de Visser M (2012). Effect of enzyme therapy and prognostic factors in 69 adults with Pompe disease: an open-label single-center study. Orphanet J Rare Dis.

[10] Kuperus E, Kruijshaar ME, Wens SCA, de Vries JM, Favejee MM, van der Meijden JC (2017). Long-term benefit of enzyme replacement therapy in Pompe disease: a 5-year prospective study. Neurology..

[11] Gungor D, Kruijshaar ME, Plug I, D'Agostino RB, Hagemans ML, van Doorn PA (2013). Impact of enzyme replacement therapy on survival in adults with Pompe disease: results from a prospective international observational study. Orphanet J Rare Dis.

[12] van der Ploeg AT, Kruijshaar ME, Toscano A, Laforet P, Angelini C, Lachmann RH (2017). European consensus for starting and stopping enzyme replacement therapy in adult patients with Pompe disease: a 10-year experience. Eur J Neurol.

[13] Harlaar L, Hogrel JY, Perniconi B, Kruijshaar ME, Rizopoulos D, Taouagh N (2019). Large variation in effects during 10 years of enzyme therapy in adults with Pompe disease. Neurology.

[14] Hagemans ML, Janssens AC, Winkel LP, Sieradzan KA, Reuser AJ, Van Doorn PA (2004). Late-onset Pompe disease primarily affects quality of life in physical health domains. Neurology.

[15] Hagemans ML, Laforet P, Hop WJ, Merkies IS, Van Doorn PA, Reuser AJ (2007). Impact of late-onset Pompe disease on participation in daily life activities: evaluation of the Rotterdam handicap scale. Neuromuscul Disord.

[16] Güngör D, Kruijshaar ME, Plug I, Rizopoulos D, Kanters TA, Wens SCA (2016). Quality of life and participation in daily life of adults with Pompe disease receiving enzyme replacement therapy: 10 years of international follow-up. J Inherit Metab Dis.

[17] van der Meijden JC, Gungor D, Kruijshaar ME, Muir AD, Broekgaarden HA, van der Ploeg AT (2015). Ten years of the international Pompe survey: patient reported outcomes as a reliable tool for studying treated and untreated children and adults with non-classic Pompe disease. J Inherit Metab Dis.

[18] Medical Research Council (1976). Aids to examination of the peripheral nervous system. Memorandum no. 45.

[19] van der Ploeg RJ, Fidler V, Oosterhuis HJ (1991). Hand-held myometry: reference values. J Neurol Neurosurg Psychiatry.

[20] American Thoracic Society Committee on Proficiency Standards for Clinical Pulmonary Function Laboratories (2002). ATS statement: guidelines for the six-minute walk test. Am J Respir Crit Care Med.

[21] Enright PL, Sherrill DL (1998). Reference equations for the six-minute walk in healthy adults. Am J Respir Crit Care Med.

[22] Quanjer PH, Tammeling GJ, Cotes JE, Pedersen OF, Peslin R, Yernault JC (1993). Lung volumes and forced ventilatory flows. Report working party standardization of lung function tests, European Community for steel and coal. Official statement of the European Respiratory Society. Eur Respir J Suppl.

[23] American Thoracic Society/European Respiratory Society (2002). ATS/ERS statement on respiratory muscle testing. Am J Respir Crit Care Med.

[24] Ware JE, Sherbourne CD (1992). The MOS 36-item short-form health survey (SF-36). I. Conceptual framework and item selection. Med Care.

[25] Aaronson NK, Muller M, Cohen PD, Essink-Bot ML, Fekkes M, Sanderman R (1998). Translation, validation, and norming of the Dutch language version of the SF-36 health survey in community and chronic disease populations. J Clin Epidemiol.

[26] Merkies IS, Schmitz PI, Van Der Meche FG, Samijn JP, Van Doorn PA (2002). Psychometric evaluation of a new handicap scale in immune-mediated polyneuropathies. Muscle Nerve.

[27] van der Beek NA, Hagemans ML, van der Ploeg AT, van Doorn PA, Merkies IS (2013). The Rasch-built Pompe-specific activity (R-PAct) scale. Neuromuscul Disord.

[28] Kanters TA, Redekop K, Rutten-Van Mölken MPMH, Kruijshaar ME, Güngör D (2015). A conceptual disease model for adult Pompe disease. Orphanet J Rare Dis.

[29] Berger KI, Kanters S, Jansen JP, Stewart A, Sparks S, An Haack K (2019). Forced vital capacity and cross-domain late-onset Pompe disease outcomes: an individual patient-level data meta-analysis. J Neurol.

[30] Kempen GI, Ormel J, Brilman EI, Relyveld J (1997). Adaptive responses among Dutch elderly: the impact of eight chronic medical conditions on health-related quality of life. Am J Public Health.

[31] Ilie G, Bradfield J, Moodie L, Lawen T, Ilie A, Lawen Z (2019). The role of response-shift in studies assessing quality of life outcomes among Cancer patients: a systematic review. Front Oncol.

[32] Schwartz CE, Sprangers MA (1999). Methodological approaches for assessing response shift in longitudinal health-related quality-of-life research. Soc Sci Med.

