# Supplementary material for: Positive association between physical outcomes and patient-reported outcomes in late-onset Pompe disease: a cross sectional study
Source: Orphanet J Rare Dis. 2020 Sep 3;15:232. doi: 10.1186/s13023-020-01469-7 (PMC7469279; doi:10.1186/s13023-020-01469-7)
Supplement: Supplementary file 1 — Additional file 1. [file 13023_2020_1469_MOESM1_ESM.docx]

**WEB SUPPLEMENTAL MATERIAL FOR:**

**Positive association between physical outcomes and patient-reported outcomes in late-onset Pompe disease: a cross sectional study**

Meng Yuan^a,b^, MSc; Eleni-Rosalina Andrinopoulou^a^, PhD; Michelle E. Kruijshaar^b^, PhD;

Laurike Harlaar^c^, MD; Ans T. van der Ploeg^b^, MD, PhD; Dimitris Rizopoulos^a^, PhD; Nadine A.M.E. van der Beek^c^, MD, PhD.

**Table 1 Patient number for each pair of outcomes**

|  | Patient number | Median time pre-ERT in years (range) |
| --- | --- | --- |
| FVC upright with SF-36 PCS | 99 | 0.09 ( 0, 1.899 ) |
| FVC upright with SF-36 MCS | 99 | 0.09 ( 0, 1.899 ) |
| FVC upright with RHS | 108 | 0.11 ( 0, 1.899 ) |
| FVC upright with R-PAct | 99 | 0.082 ( 0, 1.899 ) |
| FVC supine with SF-36 PCS | 88 | 0.099 ( 0, 1.899 ) |
| FVC supine with SF-36 MCS | 88 | 0.099 ( 0, 1.899 ) |
| FVC supine with RHS | 96 | 0.112 ( 0, 1.899 ) |
| FVC supine with R-PAct | 91 | 0.107 ( 0, 1.899 ) |
| HHD with SF-36 PCS | 86 | 0.126 ( 0, 1.858 ) |
| HHD with SF-36 MCS | 86 | 0.126 ( 0, 1.858 ) |
| HHD with RHS | 96 | 0.132 ( 0, 1.841 ) |
| HHD with R-PAct | 91 | 0.121 ( 0 , 1.841 ) |
| MRC with SF-36 PCS | 101 | 0.121 ( 0, 1.899 ) |
| MRC with SF-36 MCS | 101 | 0.121 ( 0, 1.899 ) |
| MRC with RHS | 111 | 0.121 ( 0, 1.899 ) |
| MRC with R-PAct | 99 | 0.112 ( 0, 1.899 ) |
| 6MWT with SF-36 PCS | 48 | 0.055 ( 0, 1.841 ) |
| 6MWT with SF-36 MCS | 48 | 0.055 ( 0, 1.841 ) |
| 6MWT with RHS | 49 | 0.044 ( 0, 1.841 ) |
| 6MWT with R-PAct | 53 | 0.079 ( 0, 1.841 ) |

**Table 2 The results of F test for comparing linear and non-linear models**

|  | P-value |
| --- | --- |
| FVC supine with SF-36 PCS | 0.378 |
| FVC supine with SF-36 MCS | 0.051 |
| FVC supine with RHS | 0.780 |
| FVC supine with R-PAct | 0.975 |
| FVC upright with SF-36 PCS | 0.310 |
| FVC upright with SF-36 MCS | 0.309 |
| FVC upright with RHS | 0.101 |
| FVC upright with R-PAct | 0.448 |
| HHD with SF-36 PCS | 0.279 |
| HHD with SF-36 MCS | 0.816 |
| HHD with RHS | 0.295 |
| HHD with R-PAct | *0.007* |
| MRC with SF-36 PCS | *0.023* |
| MRC with SF-36 MCS | 0.174 |
| MRC with RHS | 0.668 |
| MRC with R-PAct | 0.689 |
| 6MWT with SF-36 PCS | 0.584 |
| 6MWT with SF-36 MCS | 0.120 |
| 6MWT with RHS | 0.563 |
| 6MWT with R-PAct | 0.116 |

**Fig. 1 The scatter plots of MRC and PCS score, HHD and R-PAct score with fitted linear regression line (in blue) and smoothing splines (in red)**


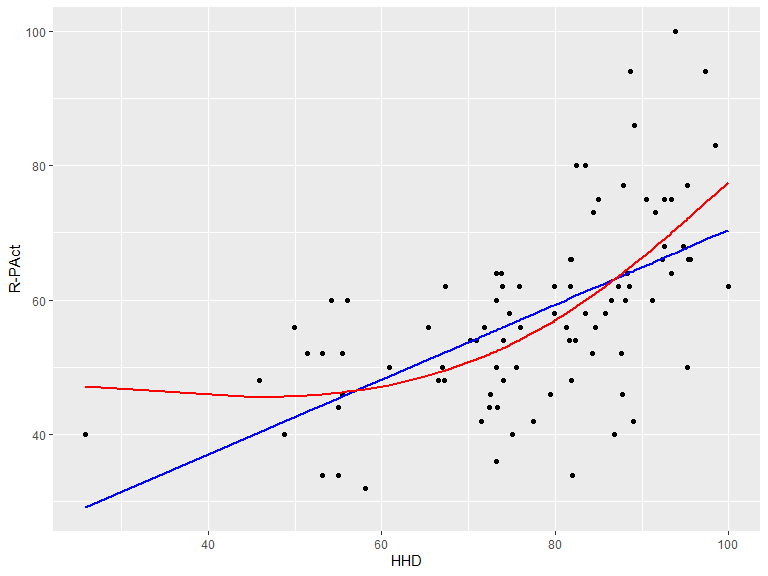

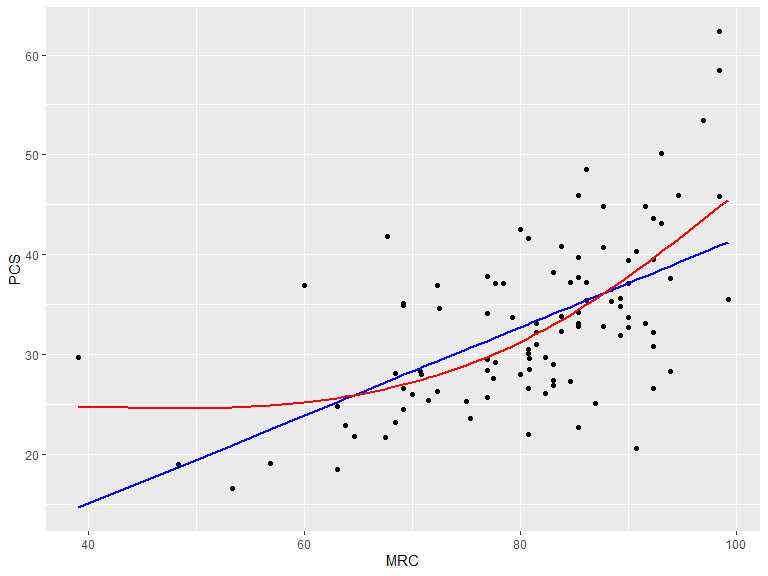


**Table 3.1 Relationship between FVC upright and PCS**

(Model: PCS = FVC upright + Sex + DiseaseDuration + Wheelchair + Ventilation)

|  | Estimates(CI) | P-value |
| --- | --- | --- |
| FVC upright | 0.178 ( 0.091 , 0.265 ) | 0.001 |
| Sex | -1.963 ( -5.003 , 1.076 ) | 1.000 |
| DiseaseDuration | -0.044 ( -0.189 , 0.101 ) | 1.000 |
| Wheelchair | -2.566 ( -5.886 , 0.754 ) | 1.000 |
| Ventilator | 0.981 ( -3.484 , 5.446 ) | 1.000 |

**Table 3.2 Relationship between FVC upright and MCS**

(Model: MCS = FVC upright + Sex + DiseaseDuration + Wheelchair + Ventilation)

|  | Estimates(CI) | P-value |
| --- | --- | --- |
| FVC upright | -0.066 ( -0.19 , 0.058 ) | 1 |
| Sex | 2.952 ( -1.372 , 7.277 ) | 1 |
| DiseaseDuration | -0.035 ( -0.242 , 0.172 ) | 1 |
| Wheelchair | 0.312 ( -4.412 , 5.036 ) | 1 |
| Ventilator | -0.584 ( -6.937 , 5.769 ) | 1 |

**Table 3.3 Relationship between FVC upright and RHS**

(Model: RHS = FVC upright + Sex + DiseaseDuration + Wheelchair + Ventilation)

|  | Estimates(CI) | P-value |
| --- | --- | --- |
| FVC upright | 0.147 ( 0.107 , 0.186 ) | <0.001 |
| Sex | -1.918 ( -3.299 , -0.538 ) | 0.138 |
| DiseaseDuration | -0.029 ( -0.096 , 0.038 ) | 1.000 |
| Wheelchair | -4.069 ( -5.599 , -2.539 ) | <0.001 |
| Ventilator | 0.023 ( -1.953 , 1.998 ) | 1.000 |

**Table 3.4 Relationship between FVC upright and R-PAct**

(Model: R-PAct = FVC upright + Sex + DiseaseDuration + Wheelchair + Ventilation)

|  | Estimates(CI) | P-value |
| --- | --- | --- |
| FVC upright | 0.359 ( 0.223 , 0.494 ) | <0.001 |
| Sex | -4.543 ( -9.251 , 0.164 ) | 1.000 |
| DiseaseDuration | -0.155 ( -0.377 , 0.066 ) | 1.000 |
| Wheelchair | -11.083 ( -16.09 , -6.077 ) | 0.001 |
| Ventilator | 0.876 ( -5.982 , 7.733 ) | 1.000 |

**Table 3.5 Relationship between FVC supine and PCS**

(Model: PCS = FVC supine + Sex + DiseaseDuration + Wheelchair + Ventilation)

|  | Estimates(CI) | P-value |
| --- | --- | --- |
| FVC supine | 0.121 ( 0.03 , 0.212 ) | 0.061 |
| Sex | -2.1 ( -5.556 , 1.356 ) | 1.000 |
| DiseaseDuration | -0.089 ( -0.25 , 0.071 ) | 1.000 |
| Wheelchair | -2.717 ( -6.418 , 0.983 ) | 1.000 |
| Ventilator | -1.898 ( -7.481 , 3.685 ) | 1.000 |

**Table 3.6 Relationship between FVC supine and MCS**

(Model: MCS = FVC supine + Sex + DiseaseDuration + Wheelchair + Ventilation)

|  | Estimates(CI) | P-value |
| --- | --- | --- |
| FVC supine | -0.099 ( -0.224 , 0.026 ) | 0.594 |
| Sex | 3.045 ( -1.675 , 7.765 ) | 1.000 |
| DiseaseDuration | -0.028 ( -0.247 , 0.191 ) | 1.000 |
| Wheelchair | -0.125 ( -5.18 , 4.93 ) | 1.000 |
| Ventilator | 0.013 ( -7.613 , 7.639 ) | 1.000 |

**Table 3.7 Relationship between FVC supine and RHS**

(Model: RHS = FVC supine + Sex + DiseaseDuration + Wheelchair + Ventilation)

|  | Estimates(CI) | P-value |
| --- | --- | --- |
| FVC supine | 0.088 ( 0.046 , 0.129 ) | 0.001 |
| Sex | -1.722 ( -3.299 , -0.145 ) | 0.622 |
| DiseaseDuration | -0.035 ( -0.109 , 0.039 ) | 1.000 |
| Wheelchair | -4.226 ( -5.908 , -2.544 ) | <0.001 |
| Ventilator | 0.35 ( -2.082 , 2.782 ) | 1.000 |

**Table 3.8 Relationship between FVC supine and R-PAct**

(Model: R-PAct = FVC supine + Sex + DiseaseDuration + Wheelchair + Ventilation)

|  | Estimates(CI) | P-value |
| --- | --- | --- |
| FVC supine | 0.245 ( 0.124 , 0.366 ) | 0.002 |
| Sex | -4.865 ( -10.003 , 0.273 ) | 1.000 |
| DiseaseDuration | -0.202 ( -0.439 , 0.034 ) | 1.000 |
| Wheelchair | -10.379 ( -15.721 , -5.036 ) | 0.003 |
| Ventilator | 0.238 ( -7.68 , 8.155 ) | 1.000 |

**Table 3.9 Relationship between HHD and PCS**

(Model: PCS = HHD + Sex + DiseaseDuration + Wheelchair + Ventilation)

|  | Estimates(CI) | P-value |
| --- | --- | --- |
| HHD | 0.186 ( 0.053 , 0.319 ) | 0.046 |
| Sex | -0.526 ( -3.931 , 2.88 ) | 1.000 |
| DiseaseDuration | -0.045 ( -0.207 , 0.117 ) | 1.000 |
| Wheelchair | -2.915 ( -6.763 , 0.933 ) | 1.000 |
| Ventilator | -2.553 ( -7.827 , 2.721 ) | 1.000 |

**Table 3.10 Relationship between HHD and MCS**

(Model: MCS = HHD + Sex + DiseaseDuration + Wheelchair + Ventilation)

|  | Estimates(CI) | P-value |
| --- | --- | --- |
| HHD | 0.025 ( -0.15 , 0.2 ) | 1 |
| Sex | 3.231 ( -1.257 , 7.719 ) | 1 |
| DiseaseDuration | 0.022 ( -0.192 , 0.235 ) | 1 |
| Wheelchair | 1.479 ( -3.592 , 6.549 ) | 1 |
| Ventilator | 0.432 ( -6.517 , 7.382 ) | 1 |

**Table 3.11 Relationship between HHD and RHS**

(Model: RHS = HHD + Sex + DiseaseDuration + Wheelchair + Ventilation)

|  | Estimates(CI) | P-value |
| --- | --- | --- |
| HHD | 0.097 ( 0.034 , 0.16 ) | 0.022 |
| Sex | -0.968 ( -2.553 , 0.616 ) | 1.000 |
| DiseaseDuration | -0.03 ( -0.108 , 0.048 ) | 1.000 |
| Wheelchair | -3.806 ( -5.634 , -1.979 ) | 0.001 |
| Ventilator | -1.479 ( -3.773 , 0.815 ) | 1.000 |

**Table 3.12 Relationship between HHD and R-PAct**

(Model: R-PAct = HHD + Sex + DiseaseDuration + Wheelchair + Ventilation)

|  | Estimates(CI) | P-value |
| --- | --- | --- |
| HHD | 0.375 ( 0.187 , 0.563 ) | 0.002 |
| Sex | -0.613 ( -5.519 , 4.293 ) | 1.000 |
| DiseaseDuration | -0.154 ( -0.387 , 0.078 ) | 1.000 |
| Wheelchair | -7.878 ( -13.671 , -2.086 ) | 0.116 |
| Ventilator | -4.769 ( -11.501 , 1.963 ) | 1.000 |

**Table 3.13 Relationship between MRC and PCS**

(Model: PCS = MRC + Sex + DiseaseDuration + Wheelchair + Ventilation)

|  | Estimates(CI) | P-value |
| --- | --- | --- |
| MRC | 0.357 ( 0.191 , 0.523 ) | 0.001 |
| Sex | -0.856 ( -3.762 , 2.051 ) | 1.000 |
| DiseaseDuration | 0 ( -0.147 , 0.148 ) | 1.000 |
| Wheelchair | -2.601 ( -6.006 , 0.805 ) | 1.000 |
| Ventilator | -1.687 ( -5.589 , 2.214 ) | 1.000 |

**Table 3.14 Relationship between MRC and MCS**

(Model: MCS = MRC + Sex + DiseaseDuration + Wheelchair + Ventilation)

|  | Estimates(CI) | P-value |
| --- | --- | --- |
| MRC | -0.11 ( -0.346 , 0.126 ) | 1 |
| Sex | 2.397 ( -1.73 , 6.524 ) | 1 |
| DiseaseDuration | -0.016 ( -0.225 , 0.193 ) | 1 |
| Wheelchair | 0.904 ( -3.931 , 5.74 ) | 1 |
| Ventilator | -0.611 ( -6.152 , 4.929 ) | 1 |

**Table 3.15 Relationship between MRC and RHS**

(Model: RHS = MRC + Sex + DiseaseDuration + Wheelchair + Ventilation)

|  | Estimates(CI) | P-value |
| --- | --- | --- |
| MRC | 0.226 ( 0.139 , 0.313 ) | <0.001 |
| Sex | -0.706 ( -2.201 , 0.789 ) | 1.000 |
| DiseaseDuration | -0.036 ( -0.112 , 0.04 ) | 1.000 |
| Wheelchair | -3.626 ( -5.389 , -1.862 ) | 0.001 |
| Ventilator | -2.48 ( -4.459 , -0.501 ) | 0.291 |

**Table 3.16 Relationship between MRC and R-PAct**

(Model: R-PAct = MRC + Sex + DiseaseDuration + Wheelchair + Ventilation)

|  | Estimates(CI) | P-value |
| --- | --- | --- |
| MRC | 1.097 ( 0.781 , 1.413 ) | <0.001 |
| Sex | 1.28 ( -3.28 , 5.839 ) | 1.000 |
| DiseaseDuration | -0.14 ( -0.358 , 0.079 ) | 1.000 |
| Wheelchair | -4.149 ( -9.905 , 1.607 ) | 1.000 |
| Ventilator | -6.166 ( -12.166 , -0.166 ) | 0.794 |

**Table 3.17 Relationship between 6MWT and PCS**

(Model: PCS = 6MWT + Sex + DiseaseDuration + Wheelchair + Ventilation)

|  | Estimates(CI) | P-value |
| --- | --- | --- |
| 6MWT | 0.246 ( 0.118 , 0.374 ) | 0.004 |
| Sex | 0.099 ( -4.344 , 4.543 ) | 1.000 |
| DiseaseDuration | -0.02 ( -0.243 , 0.202 ) | 1.000 |
| Wheelchair | 0.017 ( -5.449 , 5.483 ) | 1.000 |
| Ventilator | -4.244 ( -11.301 , 2.813 ) | 1.000 |

**Table 3.18 Relationship between 6MWT and MCS**

(Model: MCS = 6MWT + Sex + DiseaseDuration + Wheelchair + Ventilation)

|  | Estimates(CI) | P-value |
| --- | --- | --- |
| 6MWT | -0.02 ( -0.192 , 0.151 ) | 1 |
| Sex | 4.622 ( -1.331 , 10.575 ) | 1 |
| DiseaseDuration | -0.074 ( -0.373 , 0.224 ) | 1 |
| Wheelchair | 4.763 ( -2.56 , 12.087 ) | 1 |
| Ventilator | -5.521 ( -14.976 , 3.934 ) | 1 |

**Table 3.19 Relationship between 6MWT and RHS**

(Model: RHS = 6MWT + Sex + DiseaseDuration + Wheelchair + Ventilation)

|  | Estimates(CI) | P-value |
| --- | --- | --- |
| 6MWT | 0.111 ( 0.051 , 0.17 ) | 0.005 |
| Sex | -1.567 ( -3.665 , 0.531 ) | 1.000 |
| DiseaseDuration | 0.037 ( -0.069 , 0.143 ) | 1.000 |
| Wheelchair | -2.831 ( -5.445 , -0.216 ) | 0.448 |
| Ventilator | -3.267 ( -6.35 , -0.184 ) | 0.729 |

**Table 3.20 Relationship between 6MWT and R-PAct**

(Model: R-PAct = 6MWT + Sex + DiseaseDuration + Wheelchair + Ventilation)

|  | Estimates(CI) | P-value |
| --- | --- | --- |
| 6MWT | 0.348 ( 0.147 , 0.549 ) | 0.01 |
| Sex | -6.029 ( -13.335 , 1.277 ) | 1.00 |
| DiseaseDuration | -0.124 ( -0.446 , 0.198 ) | 1.00 |
| Wheelchair | -5.857 ( -14.557 , 2.844 ) | 1.00 |
| Ventilator | -1.6 ( -12.386 , 9.186 ) | 1.00 |

**Table 4 Association between physical outcomes and PROMs**

|  | R-PAct score | P-value | RHS score | P-value | SF-36 PCS score | P-value | SF-36 MCS score | P-value |
| --- | --- | --- | --- | --- | --- | --- | --- | --- |
| FVC supine | 0.396 ( 0.203 , 0.588 ) | 0.002 | 0.387 ( 0.207 , 0.567 ) | 0.001 | 0.312 ( 0.08 , 0.544 ) | 0.061 | -0.207 ( -0.464 , 0.05 ) | 0.594 |
| FVC upright | 0.485 ( 0.304 , 0.666 ) | <0.001 | 0.56 ( 0.41 , 0.71 ) | <0.001 | 0.47 ( 0.244 , 0.696 ) | 0.001 | -0.145 ( -0.411 , 0.122 ) | 1.000 |
| HHD sum score | 0.386 ( 0.196 , 0.577 ) | 0.002 | 0.29 ( 0.105 , 0.474 ) | 0.022 | 0.323 ( 0.096 , 0.55 ) | 0.046 | 0.037 ( -0.22 , 0.294 ) | 1.000 |
| MRC sum score | 0.595 ( 0.426 , 0.764 ) | <0.001 | 0.423 ( 0.262 , 0.583 ) | <0.001 | 0.47 ( 0.254 , 0.686 ) | 0.001 | -0.126 ( -0.393 , 0.14 ) | 1.000 |
| 6MWT | 0.495 ( 0.217 , 0.773 ) | 0.010 | 0.485 ( 0.232 , 0.739 ) | 0.005 | 0.584 ( 0.289 , 0.878 ) | 0.004 | -0.046 ( -0.417 , 0.325 ) | 1.000 |

^Each cell represents the result of one linear regression model. Standardized regression coefficients are calculated to ensure the comparability of different models. P-value is the adjusted p-value by the Holm method.^
